# Insula and Amygdala Atrophy Are Associated With Functional Impairment in Subjects With Presbycusis

**DOI:** 10.3389/fnagi.2020.00102

**Published:** 2020-04-28

**Authors:** Chama Belkhiria, Rodrigo C. Vergara, Simón San Martin, Alexis Leiva, Melissa Martinez, Bruno Marcenaro, Maricarmen Andrade, Paul H. Delano, Carolina Delgado

**Affiliations:** ^1^Neuroscience Department, Facultad de Medicina, Universidad de Chile, Santiago, Chile; ^2^Kinesiology Department, Facultad de Artes y Educación Física, Universidad Metropolitana de Ciencias de la Educación, Santiago, Chile; ^3^Neurology and Neurosurgery Department, Hospital Clínico de la Universidad de Chile, Santiago, Chile; ^4^Internal Medicine Department, Clínica Universidad de los Andes, Santiago, Chile; ^5^Otolaryngology Department, Hospital Clínico de la Universidad de Chile, Santiago, Chile; ^6^Centro Avanzado de Ingeniería Eléctrica y Electrónica, AC3E, Universidad Técnica Federico Santa María, Valparaíso, Chile; ^7^Biomedical Neuroscience Institute, Facultad de Medicina, Universidad de Chile, Santiago, Chile

**Keywords:** dementia, cochlear dysfunction, apathy, activities of daily living, behavioral impairment, presbycusis, insula atrophy

## Abstract

Hearing loss is an important risk factor for dementia. However, the mechanisms that relate these disorders are still unknown. As a proxy of this relationship, we studied the structural brain changes associated with functional impairment in activities of daily living in subjects with age related hearing loss, or presbycusis. One hundred eleven independent, non-demented subjects older than 65 years recruited in the ANDES cohort were evaluated using a combined approach including (i) audiological tests: hearing thresholds and cochlear function measured by pure tone averages and the distortion product otoacoustic emissions respectively; (ii) behavioral variables: cognitive, neuropsychiatric, and functional impairment in activities of daily living measured by validated questionnaires; and (iii) structural brain imaging—assessed by magnetic resonance imaging at 3 Tesla. The mean age of the recruited subjects (69 females) was 73.95 ± 5.47 years (mean ± SD) with an average educational level of 9.44 ± 4.2 years of schooling. According to the audiometric hearing thresholds and presence of otoacoustic emissions, we studied three groups: controls with normal hearing (*n* = 36), presbycusis with preserved cochlear function (*n* = 33), and presbycusis with cochlear dysfunction (*n* = 38). We found a significant association (*R*^2^_D_ = 0.17) between the number of detected otoacoustic emissions and apathy symptoms. The presbycusis with cochlear dysfunction group had worse performance than controls in global cognition, language and executive functions, and severe apathy symptoms than the other groups. The neuropsychiatric symptoms and language deficits were the main determinants of functional impairment in both groups of subjects with presbycusis. Atrophy of insula, amygdala, and other temporal areas were related with functional impairment, apathy, and language deficits in the presbycusis with cochlear dysfunction group. We conclude that (i) the neuropsychiatric symptoms had a major effect on functional loss in subjects with presbycusis, (ii) cochlear dysfunction is relevant for the association between hearing loss and behavioral impairment, and (iii) atrophy of the insula and amygdala among other temporal areas are related with hearing loss and behavioral impairment.

## Introduction

Age-related chronic disorders, including sensory, neurological, and mental syndromes are among the major contributors for disability (Prince et al., 2015). Dementia, or major neurocognitive disorder (McKhann et al., 2011; Sachdev et al., 2015), is usually defined as an acquired cognitive or behavioral impairment that causes functional loss which interferes with independence in everyday activities. Importantly, in low- and middle-income countries, dementia has emerged as the leading cause of disability in older people (Prince et al., 2015). Today, 50 million people live with dementia worldwide, and due to the population aging this number is projected to double every 20 years (World Alzheimer Report, 2016, ADI). In Alzheimer’s disease (AD), non-cognitive behavioral symptoms including mood, perception, and behavioral alterations are grouped as neuropsychiatric symptoms (NPS), which are important contributors for functional impairment in elders (Lyketsos et al., 2011; de Oliveira et al., 2015).

Another common cause of disability in elders is age-related hearing loss, or presbycusis. It is the third most prevalent condition in the elderly population, reaching 80% in those older than 85 years (Cruickshanks et al., 1998; Li-Korotky, 2012). Hearing loss is defined by an increase of audiogram thresholds and has recently been recognized as a new major risk factor for dementia in those older than 55 years (Livingston et al., 2017). Several longitudinal studies have demonstrated that hearing loss increases the risk of incident all-cause dementia, with a relative risk that augments linearly with the severity of hearing loss (Lin et al., 2011; Gallacher et al., 2012; Deal et al., 2016). In addition to its association with cognitive decline, hearing loss has been related with increased risk of depressive symptoms (Brewster et al., 2018; Cosh et al., 2018). Therefore, the comorbidity of disorders that are risk factors for dementia or functional disability –such as presbycusis and NPS – is very common in the elderly, as many of them are related with the aging process (Livingston et al., 2017). Accordingly, previous studies that accounted for the variables related to functional limitations in subjects with presbycusis found that functional impairment is associated with multiple conditions, such as older age, comorbidities, cognitive impairment, and more severe hearing loss (López-Torres Hidalgo et al., 2009; Solheim et al., 2011; Sogebi et al., 2015). Even in high-functioning elderly, subjective perception of hearing loss increases the risk of intellectual impairment and loss of the social role (Tomioka et al., 2015). In addition to the behavioral changes that precede dementia, recent studies suggest that biomarkers of neurodegeneration such as brain atrophy in certain areas could predict cognitive decline and dementia (ten Kate et al., 2018). Likewise, subjects with presbycusis have gray matter atrophy in the temporal lobe (Lin et al., 2014), as well as in nodes of the default mode network (Ren et al., 2018), such as the insula and the cingulate cortex. In this sense, we recently found that subjects with presbycusis and cochlear dysfunction, as evidenced by the loss of otoacoustic emissions, have atrophy of the cingulate and temporal cortices, which were related to executive dysfunction and depressive symptoms (Belkhiria et al., 2019).

In this present study, we hypothesized that the atrophy of the temporal lobe, among other brain areas related with hearing loss, will be associated not solely with neuropsychiatric symptoms and cognitive impairment, but also with functional loss in subjects with presbycusis. Our main objective was to study structural brain changes associated with functional impairment, neuropsychiatric and cognitive variables in independent non-demented subjects with presbycusis. In addition, in line with our previous work (Belkhiria et al., 2019), we evaluated whether these associations were stronger in the group with loss of otoacoustic emissions.

## Materials and Methods

### Subjects

A total of 199 adults aged ≥65 years were screened in the Auditory and Dementia study (ANDES) between 2016 and 2018 from a primary health center located in the Recoleta district in Santiago, Chile. All subjects gave written informed consent in accordance with the Declaration of Helsinki. Only 111 subjects were included in this study according to the following criteria. Inclusion criteria were to (i) be older than 65 years at the beginning of the study, (ii) possess a Mini-Mental State Examination (MMSE) score of ≥24, and (iii) demonstrate preserved functionality measured by a Pfeffer’s Functional Activities Questionnaire score of <25 (Quiroga et al., 2004). Exclusion criteria were (i) having a stroke or other symptoms of neurological disorders, (ii) having dementia, (iii) presenting psychiatric disorders, (iv) displaying other causes of hearing loss different from presbycusis, (v) using hearing aids, and (vi) other causes of significant disability, such as poor vision (Snellen test ≥50/20) or severe arthrosis. All procedures were approved by the Ethical and Scientific Committee of the Clinical Hospital of the University of Chile, permission number OAIC 752/15.

### Audiological Evaluations

Hearing thresholds were measured by an experienced audiologist with a clinical audiometer (AC40, Interacoustics^®^) in a soundproof room placed in the Otolaryngology Department of the Clinical Hospital of the University of Chile. Subjects were instructed to respond when they perceived a tone at a specific frequency and intensity. The lowest-intensity sound they could hear was registered in decibels for each frequency, including 0.125, 0.25, 0.5, 1, 2, 3, 4, 6, and 8 kHz for each ear separately. Pure tone average (PTA) hearing thresholds were calculated using 0.5, 1, 2, and 4 kHz frequencies per ear, and the PTA in the better-hearing ear was used for subsequent analyses. Subjects were classified according to the PTA of the better hearing ear into normal hearing (≤20 dB) and presbycusis (>20 dB).

Distortion product otoacoustic emissions (DPOAE) were used as a measure of the loss of outer hair cells in both ears. DPOAE measurements were performed in the same soundproof room as audiogram evaluations using a sensitive microphone (ER10C, Etymotic Research^®^) fitted to the external ear canal. The acoustic stimuli were two pure tones of different frequencies (f1 and f2) that elicited a DPOAE at 2f1–f2. The difference between the intensity of f1 and f2 tones was 10 dB (65 and 55 dB SPL), while the frequency ratio of f2/f1 was fixed at 1.22. We presented eight pairs of primary tones (f1 and f2) in each ear, eliciting eight different 2f1–f2 frequencies: 707, 891, 1,122, 1,414, 1,781, 2,244, 2,828, and 3,563 Hz. The testing time for DPOAE measurements was 180 s for each ear. The detection of the DPOAEs was based on a signal to noise amplitude criterion: a DPOAE must be at least 6 dB above the average level of the noise floor sampled at several frequencies surrounding the emission frequency.

We calculated the number of DPOAE detected in the eight different frequencies in each ear (minimum = 0, maximum = 8). For the subsequent analyses we used the sum of DPOAE obtained in both ears (minimum = 0, maximum = 16). Using a bimodal distribution of the presence of DPOAE in our data (see Belkhiria et al., 2019 for more details of the DPOAE measurements). We defined the group of subjects with presbycusis and cochlear dysfunction (PCD) as those subjects that had <4 DPOAE in the sum of both ears, while the group with preserved cochlear function (PCF) was defined as those subjects that had four or more DPOAEs in the sum of both ears. According to the hearing threshold of the better ear and cochlear function we divided the sample into three groups: (i) Control with normal hearing and preserved cochlear function: PTA < 20 dB and DPOAE in both ears ≥4; (ii) subjects with presbycusis and more preserved cochlear function: PTA ≥ 20 dB and DPOAE in both ears ≥ 4; and (iii) subjects with presbycusis and cochlear dysfunction PTA ≥ 20 dB and DPOAE in both ears <4. We used this approach because PTA and DPOAE measure different functions: audiogram PTA evaluates individual audition, while DPOAE measures loss of outer hair cells in both ears.

### Functional Loss in Activities of Daily Living

This variable was measured by the informant using the Technology-Activities of Daily Living Questionnaire (T-ADLQ) (Muñoz-Neira et al., 2012). The T-ADLQ includes advanced activities of daily living and the use of technologies that are not usually measured in other tests. It is composed of 33 items that measure ADL of different complexity, gathered in seven subscales: self-care activities, house hold care, employment and recreation, shopping and money, travel, communication, technology. Each item is rated on a 4-point scale from 0 (no problem) to 3 (no longer capable of performing the activity). Higher scores indicate greater deterioration. For each item, a rating is provided for instances in which the patient may never have performed that activity in the past, stopped the activity prior to the onset of dementia, or for which the proxy had no information (Johnson et al., 2004).

### Cognitive Assessment

A trained clinical neuropsychologist administered and scored all neuropsychological tests that were done blinded to the audiological status of the subjects. The cognitive domains were assessed using the following measures: (i) global cognitive functioning, the Chilean version of the MMSE (Quiroga et al., 2004); (ii) episodic memory, the total recall of the “Word” Chilean Spanish-version of the free and cued selective reminding test (FCSRT) (Grober et al., 1988; Delgado et al., 2016); (iii) executive function, the Frontal Assessment Battery (FAB) (Dubois et al., 2000); (iv) processing speed, the trail making test A (TMT A) (Army Individual Test Battery, 1944); (v) language, the Boston nominating test (Kaplan, 1978); and (vi) visuospatial abilities the Rey-Osterrieth complex figure test (Rey, 1959).

### Behavioral and Psychological Symptoms

NPS were measured using different questionnaires: for global measurement we used the Chilean version of the Neuropsychiatric Inventory Questionnaire (NPI-Q) (Musa et al., 2017). The NPI-Q is rated by the proxy and evaluates 12 neuropsychiatric disturbances common in dementia (Musa et al., 2017). The presence and severity of each symptom was rated from 0 to 3 on the basis of scripted questions administered to the proxy. In addition, we calculated the total NPI severity score ranking from 0 to 36. Apathy was measured using the apathy evaluation scale informant version (AES-i). It consists of an 18-item questionnaire which is rated on a 4-point Likert scale from 1 (not at all characteristic) to 4 (very characteristic). Higher scores indicate higher levels of apathy (Marin et al., 1991). Significant levels of apathy were considered two standard deviations over the total group mean score. Depression was self-rated by the subjects using the Geriatric Depression Scale (GDS), which consists of 15 Yes/No answer questions. Higher scores indicate higher levels of depression, while scores over five indicate significant depressive symptoms (Yesavage et al., 1982).

### Magnetic Resonance Imaging

Imaging data were acquired using a MAGNETOM Skyra 3 Tesla whole body MRI Scanner (Siemens Healthcare GmbHR, Erlangen, Germany) using a T1 MPRAGE sequence. Contiguous images across the entire brain were acquired with the following parameters: time echo (TE) = 232 ms, time repetition (TR) = 2,300 ms, flip angle = 8_, 26 slices, matrix = 256 _ 256, voxel size = 0.94 _ 0.94 _ 0.9 mm3. We also registered T2 weighted turbo spin echo (TSE) (4,500 TR ms, 92 TE ms) and fluid attenuated inversion recovery (FLAIR) (8,000 TR ms, 94 TE ms, 2,500 TI ms) to inspect structural abnormalities. The acquisition duration was 30 min with a total of 440 images for each subject.

### Image Processing and Analysis

The morphometric analysis was carried out using the software FreeSurfer version 6 running under Centos 6.0 (Gronenschild et al., 2012). A single Linux workstation was used for T1 weighted images analysis of individual subjects as suggested by Gronenschild et al. (2012). The FreeSurfer software processes cortical reconstruction (Fischl, 2012) through the following steps: volume registration with the Talairach atlas, bias field correction, initial volumetric labeling, non-linear alignment to the Talairach space, and final volume labeling. FreeSurfer’s standard pipeline (i.e., recon-all) was used to produce representations of the cortical parcellation. It uses both intensity and continuity information from the entire three-dimensional MRI volume in segmentation and deformation procedures. Brain volumes were normalized by the estimated total intracranial volume (eTIV) to account for variations in head size (Buckner et al., 2004). The reliability between manual tracing and automatic volume measurements has been validated (Fischl et al., 2002). All volumes were visually inspected, and if needed, edited by a trained researcher according to standard processes. T2 FLAIR MRI dataset was not used, but all surfaces were visually inspected to ensure accuracy of registration, skull stripping, segmentation, and cortical surface reconstruction. If geometric inaccuracy in boundaries between white matter, gray matter, and cerebrospinal fluid was present in the automated white matter segmentation, then manual editing was conducted. FreeSurfer morphometric procedures have been confirmed to present good test-retest reliability across scanner manufacturers and across field strengths (Han et al., 2006; Reuter et al., 2012).

After the whole-brain analysis, we selected regions of interest that have been consistently implicated in previous neuroimaging studies relating audition, cognition, and dementia (Li et al., 2017; Ren et al., 2018; Xu X.-M. et al., 2019). To define ROI’s, we used the Desikan-Killiany atlas, a gyrus-based atlas (Desikan et al., 2006) that has been commonly employed to explore cortical morphometry (Fornito et al., 2013; Jalbrzikowski et al., 2013).

### Regions of Interest (ROIs)

Considering that our study explores audition, cognition, NPS and functional impairment, we looked for the main cortical and subcortical brain areas responsible for the following functions in the literature: (i) temporal cortex (e.g., superior, middle, and inferior gyri) was atrophied in a longitudinal hearing impairment study (Lin et al., 2011); (ii) cingulate cortex has been previously related with presbycusis (Husain, 2016; Belkhiria et al., 2019); (iii) amygdala atrophy has been related with neuropsychiatric symptoms in early AD (Poulin et al., 2011); and (iv) structural and functional changes of the insula have been reported in hearing loss patients (Yang et al., 2014; Li et al., 2017; Ren et al., 2018; Xu X.-M. et al., 2019). Other regions such as the hippocampus, thalamus and accumbens areas were included because of their early affection in AD (ten Kate et al., 2018).

### Statistical Analysis

The Statistical Package for the Social Sciences (SPSS) version 20 for Windows (IBM Corp., Armonk, NY, United States) and R project version 3.3.3 was used for statistical analysis. The associations of hearing function (PTA of the better hearing ear and DPOAE) with neuropsychiatric symptoms, cognitive performance, and functionality in ADL (T-ADLQ) were examined using partial correlations and Poisson regressions. We first adjusted a Poisson regression to then evaluate the same model against a quasi-Poisson regression. If no substantial change was observed in deviances, the Poisson model was kept. For Poisson regression models, we presented pseudo *R*^2^_D_ (Cameron and Windmeijer, 1997; Martin and Hall, 2016). We then analyzed the main determinants of functional impairment by performing multiple linear regression models including the T-ADLQ as the dependent variable, while demographics, cognitive tests, apathy, irritability, depression, sleeping disorders, and audiological measurements (DPOAE or PTA) were independent variables. As DPOAE and PTA were correlated, in order to avoid collinearity they were never tested simultaneously. In the case that both were significant regressors, we kept the best based on *R*^2^. All variables used in regression models were standardized by z-scores, allowing comparison among regressors’ coefficients. We further divided the whole group according to hearing status into controls, presbycusis with more preserved cochlear function and presbycusis with cochlear dysfunction according to the number of detectable DPOAE (as explained previously in the DPOAE method section). Cognitive, neuropsychiatric, auditory, and brain volume variables were compared between the three groups (controls, PCF, and PCD) using ANCOVA with gender, age, and years of education as covariates using Tukey *post hoc* tests for specific comparisons. Partial correlations were run between measures of functional impairment and brain volume values. A *p*-value < 0.05 was considered significant. All results are reported without multiple comparison corrections between tests. However, in order to limit false positive results, we included a multiple comparison correction between tests. We also corrected by the total number of statistical tests used using a Holm-Bonferroni procedure (Holm, 1979). This correction did not include *post hoc* and regressor tests.

## Results

### Demographic Characteristics and Hearing Status

The mean age of the recruited subjects (*n* = 111, 69 female) was 73.95 ± 5.47 years (mean ± SD) with an average educational level of 9.44 ± 4.2 years of schooling. According to the audiometric hearing thresholds, 40 normal-hearing subjects and 71 with presbycusis participated. The average number of detected DPOAE as sum of both ears was 6.95 ± 5.56, which had a non-normal, bimodal distribution (W = 0.89, *p <* 0.001). Based on PTA and DPOAEs results, the sample was stratified into three groups: (i) controls (36 subjects), (ii) presbycusis with more preserved cochlear function (PCF, 33 subjects), and (iii) presbycusis with cochlear dysfunction (PCD, 38 subjects). Four subjects from the control group who did not fit this classification criteria were excluded from the study. A summary of demographic data, including age, sex, education, hearing level, and cardiovascular risk factors is presented in Table 1.

**TABLE 1:** Demographic description of the ANDES cohort.

Characteristic	Cohort (*n* = 111)
Age, mean (*SD*)	73.95 (5.47)
Sex, *n* (%) Female	69 (62.16)
Education, mean years (*SD*)	9.44 (4.27)
Hearing threshold, mean dB (*SD*)	26.59 (12.20)
DPOAEs, mean number (*SD*)	6.95 (5.56)
**Hearing loss and cochlear status category, *n* (%)**	
Normal hearing with preserved cochlear function	36 (32.43)
Presbycusis with preserved cochlear function	33 (29.72)
Presbycusis with cochlear dysfunction	38 (34.23)
Hypertension, *n* (%)	43 (38.73)
Smoking, *n* (%)	21 (18.91)
Diabetes, *n* (%)	29 (26.12)
Hearing aid use, *n* (%)	0 (0.0)

### Correlations Between Audiological, Demographic, and Behavioral Variables in the Entire Group

**FIGURE 1:**
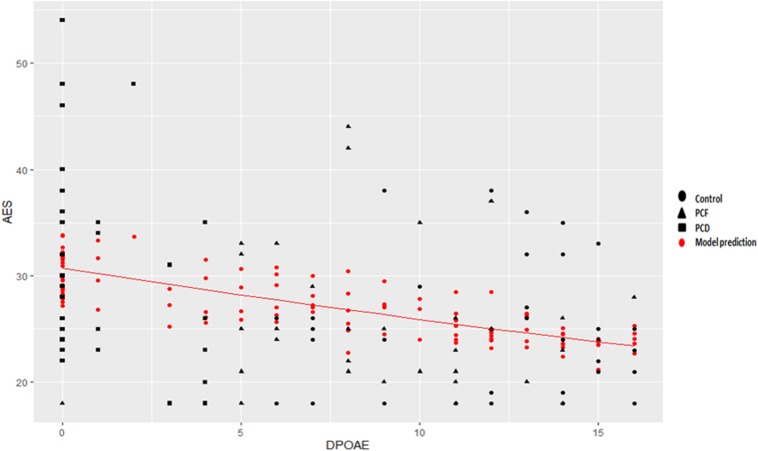
Poisson regression between apathy symptoms and cochlear function. A significant Poisson regression, including age, gender, and education, was obtained between apathy and the number of detectable DPOAE in both ears [β = –0.015 (±0.003), *z* = –4.16, *p* = 3.18e-05^#^, *R*^2^_D_ = 0.17]. Black indicates actual observations. Red dots correspond to the model’s prediction, correcting for age, gender, and years of education. The Poisson regression including only DPOAE, without age, gender, or years of education, accounts for 11% of variance depicted as a red line [β = –0.016 (±0.003), *z* = –4.99, –0.28, *p* = 5.8e-07^#^, *R*^2^_D_ = 0.11].

PTA and DPOAE numbers were significantly correlated (*r* = –0.74, *p <* 0.001), and both were correlated with age (*r* = 0.36, *p <* 0.001 and *r* = –0.41, *p <* 0.001, respectively). When age, gender, and education were included, PTA and DPOAE were correlated with the nomination score (*r* = –0.29, *p <* 0.01 and *r* = 0.25, *p <* 0.01, respectively) and with executive functions (*r* = –0.3, *p <* 0.01 and *r* = 0.3, *p <* 0.01, respectively). There was a significant association obtained through multiple Poisson regressions between apathy and the number of detectable DPOAE in both ears (β = –0.015 (±0.003), *z* = –4.16, *p* = 3.18e-05^#^, *R*^2^_D_ = 0.17) including age, gender, and years of education (Figure 1), while the same model, substituting PTA for DPOAE, showed 0.12% of variance [β = –0.003 (±0.001), *z* = 2.35, *p* = 0.01, *R*^2^_D_ = 0.12]. When performing a Poisson regression with T-ADQL as the dependent variable and DPOAE and other control variables (age, gender, and years of education), again DPOAE was a significant regressor [β = –0.025 (± 0.006), *z* = –4.05, *p* = 5.09e-05^#^, *R*^2^_D_ = 0.15]. When performing the same analysis with PTA instead of DPOAE, PTA was not a significant regressor.

### Comparison of Behavioral Variables Across Hearing Status Groups

The analysis of the neuropsychiatric symptoms showed that most subjects (72%) have at least one neuropsychiatric symptom. The most common neuropsychiatric symptoms were sleeping disorders (35%), irritability (32%), depressive symptoms (30%), and apathy (22%). Among these NPS, only the apathy severity scored by the AES-i showed significant differences between the three groups, with more severe apathy symptoms in the presbycusis and cochlear dysfunction groups [*F*_(2, 107)_ = 6.03, *p* = 0.003]. No difference was found in the depressive symptoms score [*F*_(2, 107)_ = 0.24, *p* = 0.78].

When comparing cognitive tests between the three groups (Table 2), the group of presbycusis with cochlear dysfunction showed the worst performance for global cognition [*F*_(2, 106)_ = 5.2, *p* = 0.007], executive function [*F*_(2, 106)_ = 8.89, *p* = 0.0002] and nomination [*F*_(2, 106)_ = 3.53, *p* = 0.032]. In the entire sample, the mean percentage of functional impairment in ADL was 9.8 ± 8 (mean ± SD) which is considered a low value. No significant differences were observed for the percentage of functional impairment between the three groups [*F*_(2, 107)_ = 2.722, *p* = 0.07].

**TABLE 2:** Demographic, audiological, and cognitive comparisons across hearing status groups.

	Controls	PCF	PCD	F or χ^2^	*P*
	(*n* = 36)	(*n* = 33)	(*n* = 38)		
Age (years)	71.22 ± 4.9	73.64 ± 5.66^€^	76.73 ± 4.6^¥^	11.27	3.77E-05**^#^
Education (years)	9.72 ± 3.74	9.42 ± 4.43	9.94 ± 4.24	0.138	0.871
Sex, Female (%)	27 (75%)	20 (60%)	19 (50%)	4.912	0.086
DPOAEs (number)	12.11 ± 3.28	9.09 ± 2.87^€^	0.92 ± 1.49^¥$^	182.9	<2E-16**^#^
PTA (dB)	15.05 ± 3.6	26.27 ± 4.48^€^	39.21 ± 9.92^¥$^	119.3	<2E-16**^#^
Global cognition (MMSE)	28.26 ± 1.3	28.55 ± 0.86	27.60 ± 1.46^$^	5.207	0.007*
Executive functions (FAB)	14.52 ± 1.89	13.48 ± 2.41	12.51 ± 2.38^$^	8.891	0.0002*^#^
Visuospatial capacities (Rey figure)	29.33 ± 6.01	29.88 ± 5.42	30.43 ± 3.42	0.553	0.57
Processing speed (TMT A)	59.52 ± 28.99	55.85 ± 27.31	73.16 ± 58.45	2.043	0.13
Episodic memory (FCSRT-total recall)	42.85 ± 8.09	43.03 ± 6.47	43.64 ± 4.52	0.455	0.107
Nomination (Boston)	25.58 ± 2.70	25.06 ± 3.03	23.75 ± 3.5^¥^	3.531	0.032*
Functional impairment (T-ADLQ)	7.94 ± 7.31	9.03 ± 7.52	12.1 ± 9.23	2.722	0.07
Neuropsychiatric symptoms (NPI-Q)	1.39 ± 1.35	1.94 ± 1.71	1.68 ± 1.87	0.941	0.394
Apathy (AES-i)	25.5 ± 5.9	25.48 ± 6.95	31.07 ± 9.14^¥$^	6.121	0.003
Depression (GDS)	3.26 ± 3.21	3.17 ± 3.21	3.71 ± 3.80	0.246	0.783

Data were analyzed using ANCOVA for comparing variables between the three groups, corrected for age, sex, and education as corresponded. Differences of sex between groups were assessed using Chi-squared test. Mean ± SD values for age, education, sex, DPOAEs, PTA, and cognitive tests are shown for controls, presbycusis with more preserved cochlear function (PCF), and presbycusis with cochlear dysfunction (PCD). *p < 0.05, **p < 0.001 for significant ANCOVA. ^¥^Significant differences when comparing PCD vs. controls; ^$^significant differences when comparing PCD vs. PCF; ^€^significant differences when comparing PCF vs. controls; ^#^if significant after correcting for all statistical tests applied.

### Determinants of Functional Impairment Across Hearing Status Groups

**FIGURE 2:**
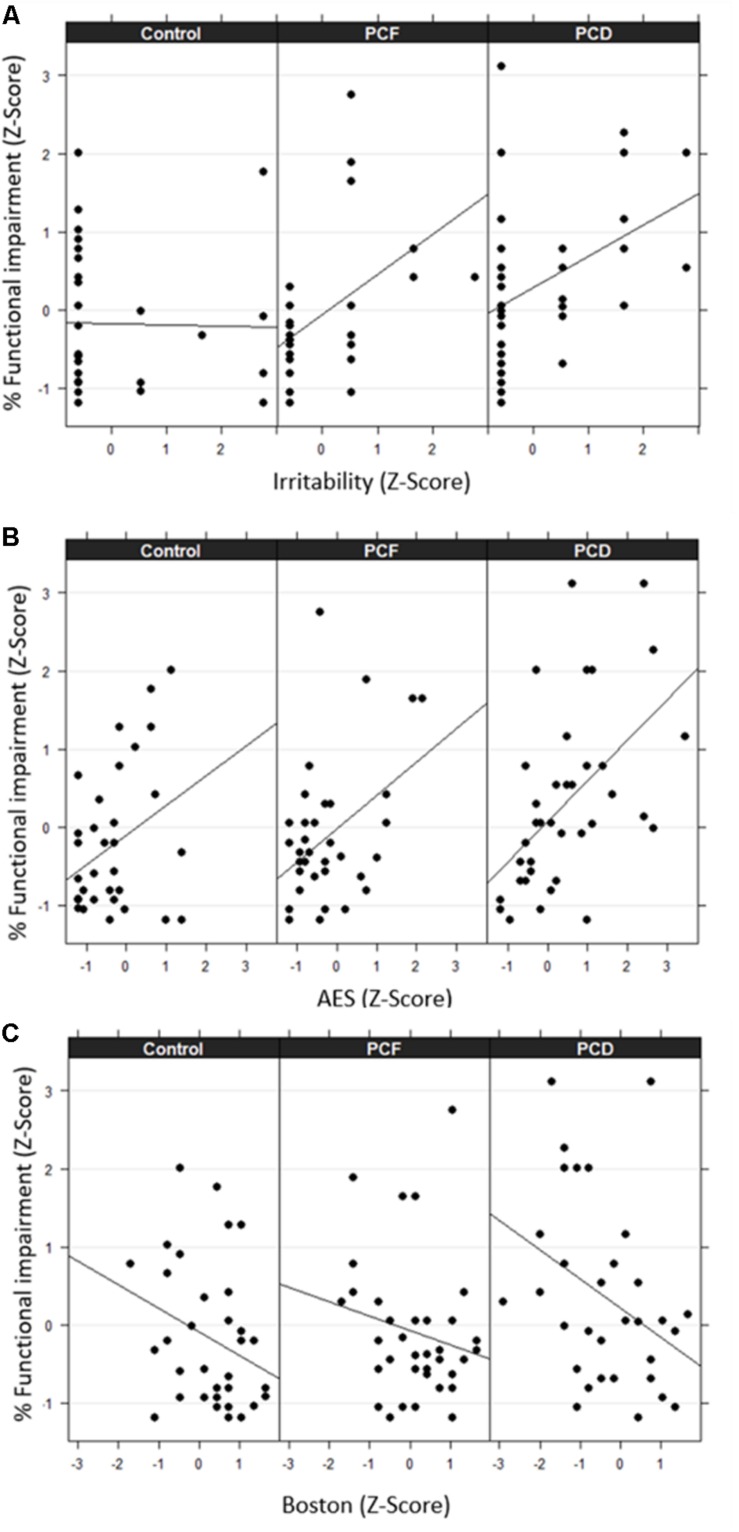
Association between percentage of functional impairment and irritability **(A)**, apathy **(B)**, and nomination **(C)** across the three groups. Values are expressed in terms of z-scores. PCF indicates presbycusis with more preserved cochlear function, while PCD indicates presbycusis with cochlear dysfunction.

We analyzed the main determinants of functional impairment by means of multiple linear regression models. We included only the apathy and irritability severity index of the NPS variables, as they were the most prevalent and severe. We also included cognitive, audiological, and demographic data. None of these variables were significant predictors of functional loss for the control group (Table 3). In contrast, for PCF [*F*_(3, 32)_ = 8.35; *p* = 0.0003^#^; *R*^2^ = 0.38] and PCD [*F*_(3, 33)_ = 9.02; *p* = 0.0001^#^; *R*^2^ = 0.40] groups, these models were significant and explained similar variance, 38 and 40% respectively (Table 3). Irritability and apathy were significant predictors for PCF and PCD functionality, while nomination was a significant predictor of functionality only in the PCD group. The DPOAE number was a significant predictor for PCF functionality. Interestingly, PTA was not a significant predictor of functional loss in any of the three groups. When observing the standardized coefficients, irritability presented a higher influence on functionality for PCF compared with PCD, while apathy presented a higher contribution to functionality for PCD compared with PCF (Table 3). The associations between irritability, apathy, nomination, and functional impairment are shown in Figure 2.

**TABLE 3:** Determinants of functional impairment in the hearing status groups.

Regressors	Control	PCF	PCD
**Regression models**			
Age	n.s.	n.s.	n.s.
Education	n.s.	n.s.	n.s.
FAB	n.s.	n.s.	n.s.
Nomination (Boston)	n.s.	n.s.	−0.35**^#^
Irritability (Severity)	n.s.	0.43**^#^	0.31*^#^
Apathy (AES-i)	n.s.	0.34*^#^	0.40**^#^
DPOAE	n.s.	−0.30*^#^	n.s.
PTA	n.s.	n.s.	n.s.
Adjusted *R*^2^	NA	0.38	0.40

Z-score standardization regression coefficients are presented. *p < 0.05, **p < 0.01, n.s. for variables tested but further pruned from the models due to none-significant results, and ^#^ if significant after correcting for all statistical tests applied. PCF, Presbycusis with more preserved cochlear function; PCD, Presbycusis with cochlear dysfunction.

### Functional Impairment and Brain Volume Changes

To examine whether the main determinants of functional loss were linked to brain volume changes, we applied partial correlations for each group. Age, gender, years of education, and estimated total intracranial volume were included as covariates. In the control and PCF groups, apathy, executive functions, and language and functional abilities were not associated with brain volume changes (not shown). In the PCD group, apathy severity (measured by AES-i), nomination and the percentage of functional loss (measured by T-ADLQ) were distinctly associated with brain volume atrophy.

**FIGURE 3:**
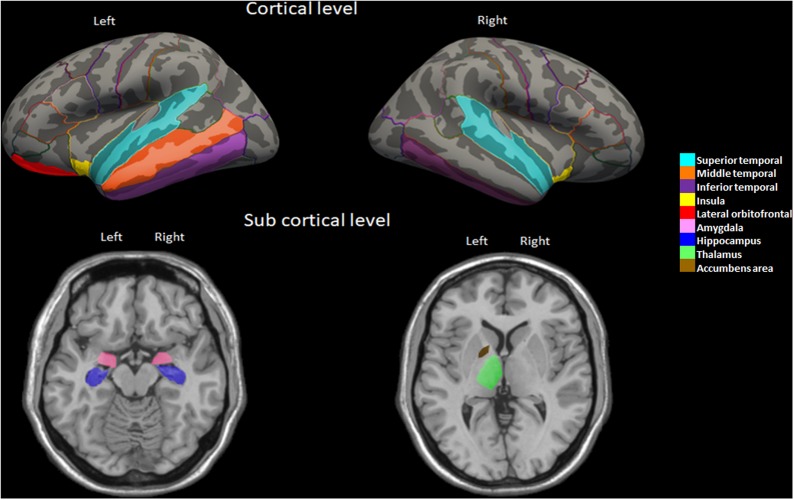
Brain regions highlighting the significant partial correlations between volume and functional impairment in the PCD group.

AES-i showed significant inverse correlations with bilateral volume of amygdala, insula, and inferior temporal gyrus. Nomination (Boston) score was directly correlated with left orbitofrontal, right thalamus and bilateral hippocampus, anterior cingulate, and insula volume (Table 4). However, the percentage of functional impairment was significantly and inversely correlated with bilateral frontotemporal regions as well as with amygdala, insula, hippocampus and nucleus accumbens volume (Table 4). These areas are represented in Figure 3.

**TABLE 4:** Partial correlations between brain volume and apathy severity, nomination score, and percentage of functional impairment in PCD group.

	Apathy	*p*	Nomination	*P*	Functional impairment	*P*	Apathy	*p*	Nomination	*p*	Functional impairment	*P*
		
	Left hemisphere						Right hemisphere					
Lateral orbitofrontal	–0.14	n.s.	0.42*	0.01	−0.34*	0.04	–0.12	n.s.	0.3	n.s.	–0.15	n.s.
Anterior cingulate	–0.08	n.s.	0.54**	0.002**	–0.21	n.s.	0.29	n.s.	0.37*	0.03	–0.24	n.s.
Posterior cingulate	–0.11	n.s.	0.15	n.s.	–0.28	n.s.	0.14	n.s.	0.28	n.s.	–0.29	n.s.
Superior temporal	–0.21	n.s.	0.17	n.s.	−0.39*	0.02	–0.11	n.s.	0.21	n.s.	−0.37*	0.03
Middle temporal	0.02	n.s.	0.21	n.s.	−0.31*	0.04	0.11	n.s.	0.12	n.s.	–0.2	n.s.
Inferior temporal	−0.34*	0.04	–0.09	n.s.	−0.39*	0.04	−0.34*	0.04	–0.09	n.s.	−0.39*	0.02
Insula	−0.3*	0.04	0.44*	0.01	−0.39*	0.02	−0.34*	0.04	0.46*	0.006	−0.46*	0.006
Amygdala	−0.31*	0.04	0.26	n.s.	−0.36*	0.03	−0.36*	0.03	0.2	n.s.	−0.51*	0.002
Hippocampus	–0.17	n.s.	0.42*	0.01	−0.46*	0.04	–0.17	n.s.	0.42*	0.01	−0.46**	0.005
Thalamus	–0.16	n.s.	–0.05	n.s.	−0.31*	0.04	–0.04	n.s.	0.35*	0.03	–0.19	n.s.
Accumbens-area	–0.13	n.s.	0.15	n.s.	−0.35*	0.04	–0.083	n.s.	0.15	n.s.	–0.27	n.s.

Results are corrected for age, gender, years of education, and estimated total intracranial volume. n.s: p > 0.05, *p < 0.05, **p < 0.01, ^#^if significant after correcting for all statistical tests applied.

## Discussion

In order to explore the mechanisms that relate presbycusis and dementia, we analyzed the structural brain changes associated with mild functional and behavioral impairment in presbycusis subjects. We found that functional loss was related with atrophy in the anterior insula and amygdala, among other temporal areas in subjects with presbycusis and cochlear dysfunction. We found a neural link relating presbycusis and mild functional impairment.

### Hearing Loss and Functional Impairment

As a first step, we presented a detailed assessment of cognitive, neuropsychiatric, and audiological variables related with functional impairment in presbycusis subjects, excluding other non-neural causes of disability. We reached a significant model that explained nearly 40% of the variability of functional loss in both presbycusis groups. The variables that were significantly related with functional loss included the NPS, nomination impairment, and cochlear dysfunction (Tables 2, 3). Surprisingly, hearing threshold as measured by PTA was not a significant predictor of functional impairment in any of the groups with presbycusis. One explanation could be due to our inclusion criterion of no use of hearing aids at recruitment. For this reason, there were relatively few subjects with moderate to severe hearing loss, which according to the World Health Organization (2017) probably had more severe functional impairment due to hearing loss.

On the other hand, the cochlear function, measured by the sum of the total DPOAEs detected in both ears, appeared to be significant predictor of functional loss in the PCF group, which was mainly comprised of mild presbycusis subjects. The lack of effect of DPOAE in the PCD group is probably caused by the low variability in the number of DPOAEs in this group (0.92 ± 1.49) compared to the PCF (9.09 ± 2.87) (mean ± SD) due to our classification criteria. Our findings suggest that the loss of DPOAE is a more sensitive measure for predicting functional impairment in subjects with mild presbycusis than PTA (average thresholds between 0.5 and 4 kHz). This is also supported by the behavioral analysis (Tables 2, 3 and Figure 2) showing that the PCD group had worse performance in cognitive tests and behavioral questionnaires compared to the PCF group.

The most important determinants of functional impairment in presbycusis were the NPS, specifically apathy and irritability. Apathy is widely defined as a marked “loss of motivation” (Marin et al., 1991; Kaji and Hirata, 2011; Kirsch-Darrow et al., 2011), which has been related with functional impairment in demented (Freels et al., 1992; Lechowski et al., 2009; Clarke et al., 2011; Rog et al., 2014; You et al., 2015; Stanton and Carson, 2016; Farias et al., 2017; Lanctôt et al., 2017; Parrao et al., 2017) and non-demented elders (Freels et al., 1992; Rog et al., 2014; van Dalen et al., 2018; Delgado et al., 2019). Considering the high prevalence of presbycusis in the elderly, it is possible that hearing loss could mediate, at least partially, the association between apathy and functional impairment described in these studies. The relationship between neuropsychiatric symptoms and sensorial loss have been reported in subjects with AD and Parkinson’s disease (Cramer et al., 2010; Hong et al., 2015), showing that apathy severity was related with olfactory dysfunction (Yu et al., 2018), suggesting that the neurodegenerative process affects the limbic system, including the olfactory system (Seligman et al., 2013). In our study, interestingly, apathy severity was significantly associated with cochlear dysfunction (loss of DPOAEs). Although it is relevant to note that we found a rather weak association (*R*^2^_D_ = 0.17), most of the variance was due to DPOAE instead of known related variables such as age and education or gender. Figure 1 showed that better cochlear function (higher DPOAE number) is associated with less severe apathy symptoms (smaller AES-i score). As far as we know, this is the first study reporting an association between apathy and a pure peripheral cause of hearing loss in non-demented subjects. Also, PTA was not associated with apathy, supporting that to some extent DPOAE complements the information obtained with PTA in regards to neurodegeneration and cognitive decline (Belkhiria et al., 2019).

### Structural Brain Changes Related With Functional Impairment in Presbycusis

In line with the behavioral results, we found significant correlations between functional impairment and atrophy of bilateral insula and several areas of the temporal cortex, including the bilateral hippocampus and amygdala in the PCD group but not in the PCF group. Also, there were significant associations with subcortical areas, including the left thalamus and the nucleus accumbens (Table 4). Among these areas, the atrophy of the bilateral insula and hippocampus was related to nomination impairment, while apathy severity was associated with atrophy of the bilateral inferior temporal cortex, insula, and amygdala (Table 4). Notably, the functional decline in the PCD group was related with the atrophy of areas beyond the core speech network (Peelle and Wingfield, 2016; Peelle, 2018). Some of these areas, such as the cingulate cortex and the insula, have been shown to be activated during speech understanding of acoustically degraded situations. During these conditions, subjects need to increase their listening effort to comprehend the acoustic information (Sharma and Glick, 2016; Xu X.-M. et al., 2019). The listening effort might increase the cognitive load, deteriorating cognitive processing of non-auditory stimuli in patients with presbycusis (Cardin, 2016; Husain, 2016). Accordingly, a recent study evaluating functional and structural MRI in hearing impaired patients (Xu X.-M. et al., 2019) showed that the functional connections between insula and other brain regions were significantly decreased and associated with emotional and cognitive dysregulation in these patients. In addition, a study in patients with presbycusis and structural MRI showed evidence that a thicker right insula was associated with better speech perception (Ren et al., 2018). In fact, the insula is an important hub in many brain networks, including the salience network, high-level cognitive control, attentional processes, language processing, and regulation of emotion and behavior (Craig, 2009; Bestelmeyer et al., 2014). Functional MRI studies showed insular activation in response to a wide variety of stimuli and paradigms involving sound detection, auditory temporal processing, and phonological processing (Ghaziri et al., 2018). Lesions of the insula or disconnections with the auditory cortex cause auditory impairment such as agnosia, musical anhedonia (Sihvonen et al., 2016) and hyperacusis (Boucher et al., 2015). The insula is known to have a direct, extensive and reciprocal functional connection with frontal areas involved in language processing, such as the prefrontal cortex and frontal operculum (Augustine, 1996; Jakab et al., 2012), which could explain its relation with nomination impairment in our study. In addition, the insula is atrophied in diseases with language impairment such as the progressive non-fluent aphasia (Nestor et al., 2003). In several neurodegenerative diseases such as AD, Parkinson’s disease, and progressive supranuclear palsy, the atrophy of insula, anterior and posterior cingulate gyri, and frontoparietal areas have been related with apathy severity (Reijnders et al., 2010; Jeong et al., 2018; Le Heron et al., 2018).

On the other hand, the amygdala receives corticofugal projections from the auditory cortex, direct projections from subcortical regions of the ascending auditory, and from other sensorial pathways (Frühholz et al., 2014; Pérez-Valenzuela et al., 2019). It has been proposed as a key component in models of tinnitus to account for emotional distress (Jastreboff, 1990). Similar to the insula, the amygdala has been demonstrated to be especially important for decision-making by triggering autonomic responses to emotional stimuli (Clark et al., 2008).

Finally, it is important to note that the brain atrophy associated with functional impairment in presbycusis subjects with cochlear dysfunction could be related with neurodegeneration, especially considering the high prevalence of AD biomarkers in cognitively normal subjects older than 65 years (Jansen et al., 2015). In fact, recent work with animals (Park et al., 2018) showed that tau levels are elevated in the hippocampus of mice with noise-induced hearing loss, while in humans with hearing loss there are elevated Tau levels in the cerebrospinal fluid associated with brain atrophy (Xu W. et al., 2019). Thus, some of the subjects involved in our study could have a preclinical state of AD and/or another neurodegenerative process. This is supported by the view that the amygdala, hippocampus, and other temporal areas are the first atrophied regions in the early stages of AD (ten Kate et al., 2018), and that atrophy of these temporal areas has been linked to functional impairment in early stages of AD (Slachevsky et al., 2019).

## Conclusion

We demonstrated that in independent non-demented subjects with presbycusis, cochlear dysfunction was related with neuropsychiatric symptoms, language dysfunction, and functional impairment in activities of daily living. These behavioral changes were associated with atrophy of the insula and amygdala, among other temporal areas. These regions regulate the sensorial, cognitive, and emotional processing necessary for decision making and behavior, suggesting a neural link between presbycusis and dementia. Cochlear dysfunction may be considered a biomarker of progressive functional impairment in presbycusis subjects.

## Limitations

Potential limitations of this study are as follows. First, we have focused only on the volumetric measurements. Future studies could investigate the possible functional connectivity between cortical and subcortical structures and their collective contributions to functional impairment and behavioral symptoms in presbycusis. Secondly, future studies might address whether low- (<0.5 kHz) and high-frequency (>4 kHz) audiogram thresholds are associated with cognitive and brain structural changes in presbycusis. Thirdly, the cross-sectional design of the present study does not permit causal inferences. Longitudinal studies are necessary to unravel the determinants and neural mechanisms of functional impairment in presbycusis. Finally, this study used a large number of statistical tests: 7 correlations, 3 linear regressions, 4 Poisson regressions, 15 ANCOVAs, and 70 partial correlations. This naturally increases the chances of detecting effects that may not be reproducible. When using cross-statistical tests *p*-value correction, many results were significant; however, others did not pass such correction. For this reason, we advise caution on the interpretation of results, and we encourage other authors to research how PTA and DPOAE may be of help in understanding dementia and neural degeneration.

## Data Availability Statement

The raw data supporting the conclusions of this manuscript will be made available by the authors, without undue reservation, to any qualified researcher.

## Ethics Statement

The studies involving human participants and all the procedures were reviewed and approved by the Ethical and Scientific Committee of the Clinical Hospital of the Universidad de Chile, permission number OAIC 752/15. The patients/participants provided their written informed consent to participate in this study.

## Author Contributions

CB analyzed the data and wrote the manuscript. RV and SS participated to analyze the data. AL, BM, MM, and MA performed the data acquisition. PD and CD performed experimental design, data analysis, and edited the manuscript.

## Conflict of Interest

The authors declare that the research was conducted in the absence of any commercial or financial relationships that could be construed as a potential conflict of interest.

## Abbreviations

ADL: activity of daily living
AES: apathy evaluation scale
DPOAE: distortion product otoacoustic emissions
PCD: presbycusis with cochlear dysfunction
PCF: preserved cochlear function
PTA: pure tone average
NPS: neuropsychiatric symptoms.
